# Classification for treatment urgency for the microphthalmia/anophthalmia spectrum using clinical and biometrical characteristics

**DOI:** 10.1111/aos.14364

**Published:** 2020-02-25

**Authors:** Annabel L.W. Groot, Maayke M.P. Kuijten, Jelmer Remmers, Asra Gilani, Daphne L. Mourits, Elke Kraal‐Biezen, Pim de Graaf, Petra J. Zwijnenburg, Annette C. Moll, Stevie Tan, Peerooz Saeed, Dyonne T. Hartong

**Affiliations:** ^1^ Department of Ophthalmology Amsterdam Orbital Center Amsterdam UMC University of Amsterdam Amsterdam Netherlands; ^2^ Department of Ophthalmology Amsterdam UMC Vrije Universiteit Amsterdam Amsterdam Netherlands; ^3^ Department of Radiology and Nuclear Medicine Amsterdam University Medical Center Amsterdam The Netherlands; ^4^ Department of Clinical Genetics Amsterdam University Medical Center Amsterdam The Netherlands

**Keywords:** orbit, child health (paediatrics), eye (globe), genetics, prosthesis

## Abstract

**Purpose:**

Current clinical classifications do not distinguish between the severity of the MICrophthalmia/Anophthalmia (MICA) spectrum with regard to treatment urgency. We aim to provide parameters for distinguishing mild, moderate and severe MICA using clinical and biometrical characteristics.

**Methods:**

We performed a single‐centre, cross‐sectional analysis of prospective cohort of 58 MICA children from September 2013 to February 2018 seen at the Amsterdam University Medical Center, The Netherlands. All patients with a visible underdeveloped globe were included. We performed full ophthalmic evaluation including horizontal palpebral fissure length, axial length by ultrasound and/or MRI measurements, paediatric and genetic evaluation. Cases were subdivided based on clinical characteristics. Biometrical data were used to calculate the relative axial length (rAL) and the relative horizontal palpebral fissure length (rHPF) compared with the healthy contralateral eye for unilateral cases.

**Results:**

In previously untreated patients, a strong correlation exists between rAL and rHPF, distinguishing between severe, moderate and mild subjects using rAL of 0–45%, 45–75% and 75%–100%, respectively. Clinical subgroups were randomly dispersed throughout the scatterplot.

**Conclusion:**

Current classifications lack clinical implications for MICA patients. We suggest measuring eyelid length and axial length to classify the severity and determine treatment strategy. The ‘severe’ group has obvious asymmetry and abnormal socket configuration for which therapy should quickly be initiated; the ‘moderately’ affected group has normal socket anatomy with a microphthalmic eye with disturbing asymmetry for which treatment should be initiated within months of development; the ‘mild’ group has a slightly smaller axial length or less obvious eyelid asymmetry for which reconstructive correction is possible, but expansive conformer treatment is unnecessary.

## Introduction

MICrophthalmia and Anophthalmia (MICA) are rare, congenital eye disorders where microphthalmia refers to the underdevelopment of an eye with small axial length, and anophthalmia refers to the absence of any structural ocular tissue. Pure anophthalmia has however proven to be extremely rare (Roos et al., 2016), since in almost all cases, there is radiologic or pathologic evidence of an ocular remnant. Cases where no ocular structure is seen during clinical examination have been referred to as ‘clinical anophthalmic cases' (Duke‐Elder 1964) but are in fact an extreme form of microphthalmia. The disorders may occur in isolation or as part of a syndrome (McLean et al. 2003; Verma & Fitzpatrick 2007; Williamson & FitzPatrick 2014). MICrophthalmia/Anophthalmia (MICA) may present uni‐ or bilaterally with abnormalities occurring in anterior segment (sclerocornea or Peters anomaly, microcornea, iris coloboma), lens (congenital cataract), vitreous (persistent fetal vasculature; PFV) and/or posterior segment (optic coloboma) (Warburg 1993; Verma & Fitzpatrick 2007; Nishina et al. 2012; Shah et al. 2012; Skalicky et al. 2013). Characteristic features of unilateral severe disease include bony orbital hypoplasia or micro‐orbitism, microblepharon and facial asymmetry, whereas characteristics of bilateral disease include sunken orbits and midfacial hypoplasia (Krastinova et al. 2001; Shaw et al. 2005; Shah et al. 2012). Reported prevalence varies from 3 per 100 000 live births for congenital anophthalmia and 14 per 100 000 for microphthalmia to a combined prevalence for both microphthalmia and anophthalmia of 4–32 per 100 000 (Verma & Fitzpatrick 2007; Roos et al. 2016).

The diagnosis may be facilitated by imaging techniques such as ultrasound or magnetic resonance imaging (MRI). Microphthalmia has radiologically been described as an eye with an axial length (AL) of 2 standard deviations below the age‐adjusted mean, typically resulting in an axial length below 21 mm for adult eyes, whereas the diagnosis of true anophthalmia is based on the absence of ocular tissue or rudimentary rest (Verma & Fitzpatrick 2007). Horizontal palpebral fissure length (HPF) measurements are commonly performed to assess treatment outcome as this measurement is an indication of facial symmetry (Wavreille et al. 2013).

Several phenotypical classifications have been described, the most recent by Skalicky (Warburg 1993; Skalicky et al. 2013). They grouped their cases based on the presence or absence of optic fissure closure defects, with the objective to investigate associations with systemic disease and inheritance patterns. Although these classification systems may be informative regarding disease origin, no distinction can be made between the severity of microphthalmia with regard to treatment strategy and urgency. Current prevailing opinion is to treat severe cases with orbital expanding prostheses to gain symmetrical bone structure and to obtain symmetrical eyelids with sufficient fornices to hold a regular ocular prosthesis. This process should be started preferably as early as tolerated (Wiese et al. 1999). This expanding treatment is not always necessary for milder cases as they can also present with only a marginally smaller and cosmetically acceptable eye. A wide range with respect to the severity of orbital bony and soft tissue underdevelopment is thus experienced, with no clear guideline for the indication and timing of expansive treatment.

We therefore present the first study to describe both clinical and biometrical characteristics of MICA and use these parameters to indicate the urgency to start treatment.

## Materials and Methods

We performed a cross‐sectional analysis of a prospective cohort from September 2013 to February 2018 on data obtained from MICA children seen at the Amsterdam University Medical Center Amsterdam, The Netherlands. Patients were seen in a multidisciplinary team including an oculoplastic surgeon, ocularist, paediatric ophthalmologist, paediatrician, clinical geneticist and specialized radiologist. The medical ethical committee of the Amsterdam University Medical Center approved the study. The parents or their guardians gave written informed consent. All patients with a visible underdeveloped globe, either presented to us as a newborn or referred to us at a later stage, were included. Only patients without given consent were excluded.

We collected medical information regarding pregnancy duration and complications, birth, other developmental or health problems, age at first visit, ethnicity, family history and previous treatment. We performed clinical evaluations (uni‐ or bilaterality, complete ophthalmic and orthoptic evaluation of both eyes, HPF measurements with a ruler) and collected information from various imaging modalities including the HPF from clinical photographs. Axial length was measured with ultrasonography and MRI defined as the distance from the anterior surface of the cornea to the fovea using the b‐scan and was performed by a specialized radiologist. Ocular findings and other developmental disorders in the head were described using orbital and cerebral MRI under general anaesthesia if children were older than 3 months. We collected data on visual acuity determined by fixation and/or preferential looking cards for younger children and picture‐based charts, tumbling E‐charts or Snellen charts for older children. In doubt of visual potential, visual evoked potentials were obtained. Visual function was classified as mild to no visual impairment, moderate to severe visual impairment or blindness as determined by the World Health Organization. We collected medical information from patient visits to the clinical geneticist or paediatrician where they obtained a full physical examination or medical information was obtained from their treating specialist. If the genetic evaluation was not yet performed by their referral hospital, the parents were offered genetic testing including but not limited to PAX6, SOX2 and OTX2, three genes identified as pivotal for eye development (Hever et al. 2006). During the course of this study, subjects were offered genetic screening performed with an ocular development ChIP‐Array, or in some cases whole exome/genome sequencing. Follow‐up visits and ultrasounds were regularly performed depending on the age and severity.

We extended the classification of Skalicky et al. by using optic fissure closure defects (OFCD) and further subdividing their ‘non‐OFCD’ category into persistent fetal vasculature (PFV), anterior segment disorders (ASD), combined ophthalmic developmental disorders (COMB; cases with disorders in multiple segments) and microphthalmia secondary to other ocular pathology (SEC). The cases where no segment distinction was possible other than ‘ocular remnant’ were classified as the ocular remnant/anophthalmia (ORA) group. The presence of cysts and growth of the affected eye on consecutive imaging were noted.

For unilateral and yet untreated cases, the relative axial length (rAL) was calculated by dividing the axial length of the affected eye by the axial length of the healthy contralateral eye. The relative horizontal palpebral fissure length (rHPF) was determined similarly. If the HPF could not reliably be measured on photographs, the clinical measurements were used. Unilateral cases regarding eye dimensions, but with a contralateral development disorder not influencing eye size, were classified as unilateral for these calculations. Biometric data of bilateral cases were assessed separately. To assess correlation between rAL and rHPF, the Spearman's rho coefficient was used; we used intraclass correlation coefficient with two‐way mixed and absolute agreement for determining variation between HPF and axial length measurements. For statistical significance of extraocular abnormalities, Pearson chi‐squared and Fisher's exact tests were used. Any growth of the affected eye was documented by difference in axial lengths between first and last visit.

## Results

Fifty‐eight cases were included, the majority of which from Caucasian descent. Forty‐four (76%) children were unilaterally and 14/58 (24%) were bilaterally affected. Of the unilaterally affected cases, 32 were microphthalmic and 12 presented with an apparently absent eye of which 6 with radiological remnant and 6 with no apparent ocular structure on MRI imaging. The bilateral group consisted of 9 true bilateral cases (5 microphthalmic, 3 with an apparently absent eye of which 1 with radiological remnant and 2 with no apparent ocular structure on ultrasonography imaging and 1 case with microphthalmia OD and ocular remnant OS); 5 cases were unilaterally affected regarding eye size, but had developmental disorders bilaterally such as anterior segment disorders and coloboma. Pregnancy details were missing from one adopted child. Eight pregnancies (14%) were complicated by: pre‐eclampsia, pregnancy‐induced hypertension, gestational diabetes, infection (of unknown origin), use of paroxetine and two mothers had preterm premature rupture of membranes of which one child had a single umbilical artery with polyhydramnion. The demographic data of the study population are summarized in Table 1.

**Table 1:** Patient characteristics.

		*n *(%)
Gender	Male	33 (57)
	Female	25 (43)
Ethnicity	Caucasian	47 (81)
	Asian	7 (12)
	Negroid	3 (5)
	Mixed	1 (2)
Laterality	Unilateral	44 (76)
	Microphthalmia	32
	Ocular remnant/anophthalmia	12
	Bilateral	14 (24)
	Microphthalmia	5
	Ocular remnant/anophthalmia	3
	An + microphtalmia	1
	Uni‐bi*	5
Subgroups	Ocular remnant/anophthalmia	16 (28)
	Optic fissure closure defect	12 (21)
	Persistent fetal vasculature	10 (17)
	Anterior segment disorder	7 (12)
	Secondary	2 (3)
	Combination	11 (19)
Pregnancy	Median 40 weeks (range 28–42)	
	Missing data	1 (2)
	Complicated	8 (14)
Age at first visit	Median 75 weeks after gestation (range newborn—15 years)	
	Missing data	1 (2)
Treatment started elsewhere	Yes	23 (40)
	No	35 (60)

* Uni‐bi: unilateral regarding eye dimensions but with a bilateral developmental disorder.

### Characteristics of different subgroups

Of the unilateral group, 39/44 (89%) had mild to no visual impairment and 5/44 (11%) children were moderately to severely impaired due to nystagmus, strabismus of the fellow eye or due to cerebral abnormalities. Of the bilateral cases, 13/14 (93%) were either visually compromised or functionally blind, and one bilateral OFCD case had normal vision with 0.8 tumbling E's. Bilateral involvement was seen in all subgroups except the PFV subgroup. For PFV, visual prognosis is relatively good compared to other groups as all contralateral eyes were unaffected except for one child with a contralateral nystagmus.

The clinical characteristics of the different subgroups are summarized in Table 2. Cysts were seen in the OFCD (*n* = 4) and COMB (*n* = 2) subgroups, of which one has been surgically removed because of its increasing size.

**Table 2:** Clinical characteristics of different subgroups.

Subgroup		Unilateral	Bilateral	Blind*	Visually impaired*	Notable
	*n*	*n* (%)	*n* (%)	*n* (%)	*n* (%)	
Ocular remnant/anophthalmia	16	12 (75)	4 (25)	4 (25)	1 (6)	All bilateral cases had SOX2 mutation (*n* = 4). High percentage extraocular abnormalities (69%). Significantly more anatomical and functional central nervous system abnormalities (8/16, p = 0.001) and motor development delay (7/16, p = 0.006)
Optic fissure closure defect	12	8 (67)	4 (33)	1 (8)	4 (33)	High percentage intraorbital cysts (*n* = 4), significantly more cardiac defects (4/12, p = 0.014)
Persistent fetal vasculature	10	10 (100)	0 (0)	0 (0)	1 (10)	All unilateral, good visual prognosis, no growth of microphthalmic eye
Anterior segment disorder	7	4 (57)	3 (43)	3 (43)	1 (14)	43% bilateral with functional blindness
Secondary	2	1 (50)	1 (50)	1 (50)	0 (0)	Secondary to (1) Coats disease and (2) retinopathy of prematurity
Combination	11	9 (82)	2 (18)	1 (9)	2 (18)	Two intraorbital cysts

* As determined by the World Health Organization.

Serious extraocular abnormalities were seen in 28/58 cases (48%) and occurred in all subgroups. For ORA cases, these were frequent and severe (11/16 cases). Significantly more anatomical and functional central nervous system abnormalities (8/16, p = 0.001 Fisher's exact) and motor development delay (7/16, p = 0.006) were seen in these children. For the OFCD subgroup, the amount of congenital cardiac defects was higher than in other groups (4/12; p = 0.014). A detailed table with all extraocular features is provided online (Table S1).

Genetic testing showed mutations in 20/47 cases, with 12 related to the eye abnormality and 8 unrelated to the clinical presentation. The most observed mutated gene was in the SOX2 gene, found in 4 of the ORA cases all bilaterally affected. No genetic abnormalities were found in 27/47 cases, and in 11 cases, no testing was performed (Table 3).

**Table 3:** Genetic results of different aetiological subgroups.

Aetiology	Case no	Genetic mutation	Size	SNP array result
Ocular remnant/anophthalmia	1	SOX2		
	21	OTX2		
	35	SOX2 de novo		
	57	SOX2 de novo		
	61	SOX2 heterozygous deletion		
Optic fissure closure defects	19	8q12.2 microdeletion (CHD7)	136 kb	(arr[hg19] 8q12.2(61,775,182‐61,911‐070)×1
	14	20q11.21 de novo duplication	600 kb	arr snp 20q11.21(SNP_A‐1968227‐>SNP_A2276843)×3
	23	11q22 deletion (YAP1)	230 kb	arr 11q22.1q22.2(102,021,286‐102,247,650)×1
	37	4(q21q31) de novo duplication	Unknown, diagnosed with karyotyping	
Anterior segment disorder	29	PTCH1 mutation		
Persistent fetal vasculature	51	18q22.3q23 deletion (including TSHZ1)	2.97 Mb	arr 18q22.3q23(70,369,002‐73,336,751)×1 dn
	55	PIK3CA		

### Biometric data

Two observers (AG and JR) independently assessed 17 HPF measurements, showing excellent agreement (Cronbach's alpha 0.979). Ultrasound measurements (72) were repeated by a specialized radiologist with two‐hour interval yielding excellent agreement (Cronbach's alpha 0.996). All axial length data are from ultrasound measurements. Axial length information was missing for three cases, and HPF was missing for one. All biometric data are summarized in Table 4.

**Table 4:** Biometrical data of microphthalmia and anophthalmia.

A. Unilateral cases								
Clinical subgroup	*n*	Axial length (AL)			*n*	Horizontal palpebral fissure (HPF)		
		Normal eye	Affected eye	Relative AL		Normal eye	Affected eye	Relative HPF
		mm (range)		% (range)		mm (range)		% (range)
Ocular remnant/anophthalmia	11	20.1 (15.6–22.8)	2.8 (0–7.0)	15% (0–40)	12	20.3 (16–24.4)	11.9 (4–17.7)	58% (23–74)
Optic fissure closure defects	10	20.1 (16.6–22.8)	15.8 (9.1–22.7)	78% (46–105)	10	20.8 (17–25)	18 (15–24)	86% (74–101)
Combination	9	20.2 (16.6–21.7)	12.0 (8.7–17.2)	60% (46–92)	10	20 (14–24.7)	17.2 (11–24)	85% (77–97)
Persistent fetal vasculature	10	19.4 (17.4–21.7)	12.2 (8.7–18)	63% (43–83)	10	19.6 (17.7–24.7)	15.9 (13.2–20)	81% (74–100)
Anterior segment disorder	5	20.1 (17.8–21.1)	17.7 (10.8–22.0)	87% (61–106)	5	20.8 (18.8–22.3)	18.5 (14.9–22.6)	88% (70–105)
Missing	3				1			

B. Bilateral cases							
Clinical subgroup	Case no	Axial length (mm)			Horizontal palpebral fissure (mm)		
		Age (weeks)	OD	OS	Age (weeks)	OD	OS
Ocular remnant/anophthalmia	1	41	6.8	3	41	7	4
Optic fissure closure defects	3	45	10.3	10.7	73	14	12
Anterior segment disorder	15	44	10.3	16.3	44	9	12
Secondary	16	538	10.8	17.4	541	22	22
Combination	54	54	17.1	14	54	20	19
Anterior segment disorder	60	49	7.7	14.3	93	11.5	15
Ocular remnant/anophthalmia	35		Nothing to measure		516	13	13
Ocular remnant anophthalmia	61		No measurements		542	10	10
Ocular remnant/anophthalmia	57		Nothing to measure		47	4	4

Of the unilateral cases, ORA eyes had a mean rAL of 15%, and the mean rAL was 78%, 60%, 63% and 87% for OFCD, COMB, PFV and ASD eyes, respectively. The average rHPF was 58% for the ORA eyes and 86%, 85%, 81% and 88% for the OFCD, COMB, PFV and ASD eyes, respectively. Six of the bilateral cases had axial length measurements; on two cases, no anatomical structure could be measured and one case visited as a second opinion but did not undergo imaging.

**Figure 1:**
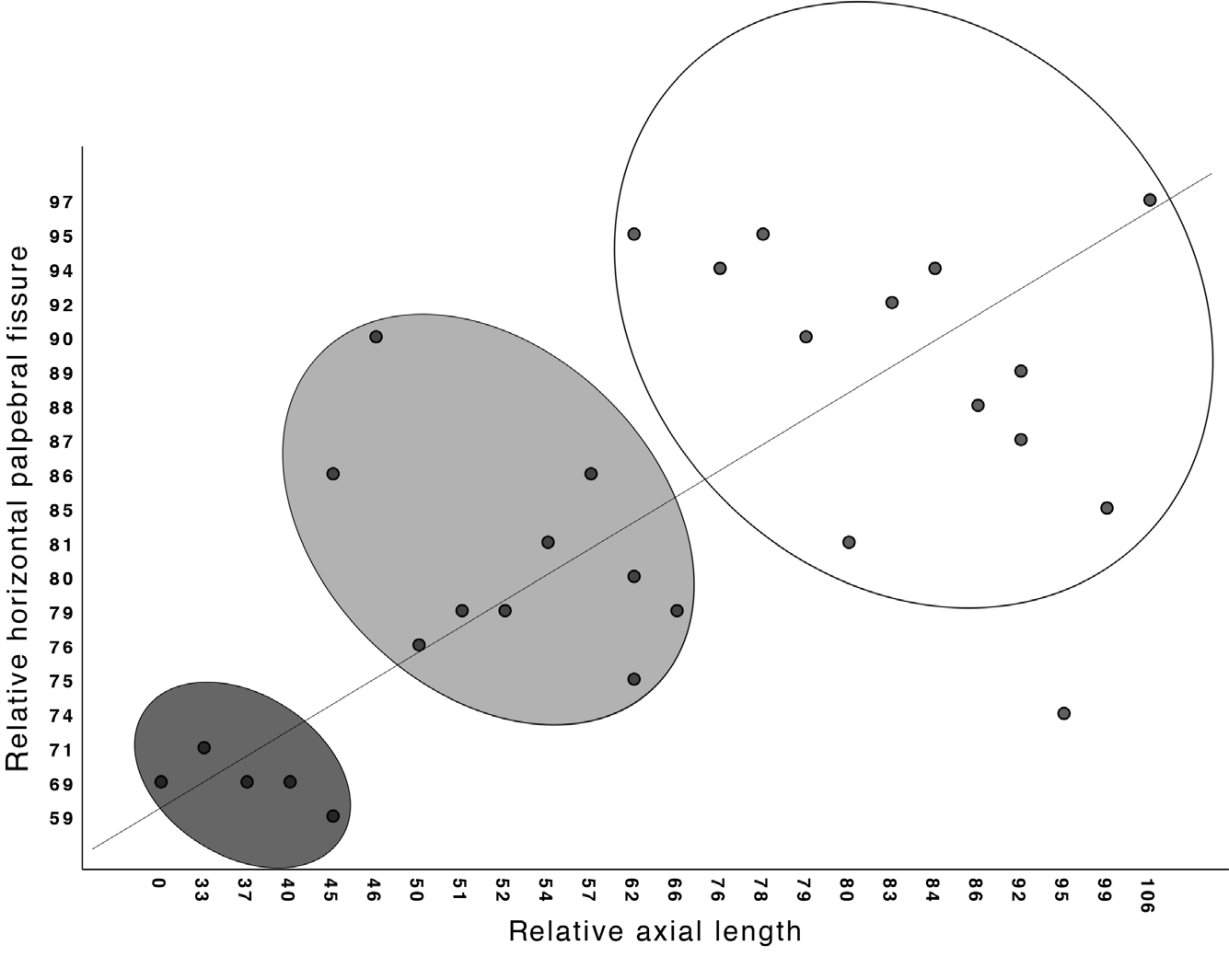
Relative axial length (rAL, X‐axis) and relative horizontal palpebral fissure (rHPF, Y‐axis) of the microphthalmic/anophthalmic eye compared with the contralateral normal eye in previously untreated cases. Severe, moderate and mild disease can be distinguished using relative axial length of the affected eye versus normal eye of 0–45%, 45–75% and 75–100%, respectively.

In previously untreated patients, we saw a strong correlation between rAL and rHPF (Spearman's coefficient 0.667; p < 0.001). When plotting rHPF against rAL, we could roughly distinguish severe, moderate and mild subjects based on the rAL: 0–45%, 45–75% and 75%–100%, respectively (Fig. 1). Growth of the microphthalmic/remnant eye, by comparing ultrasound measurements over time, could be assessed for 48 eyes of 41 cases with mean follow‐up of 2.7 years and minimum of 6 months. In unilateral cases, growth was only assessed when the contralateral healthy side showed growth. The increase in size of the microphthalmic eye was seen in 3/10 PFV eyes, 7/9 ASD eyes, 6/11 COMB eyes, 5/10 OFCD eyes and 3/5 ORA eyes in which the ocular remnant showed growth, see also Table 5. Clinical subgroups were randomly dispersed throughout the scatter plot. Five children had intraorbital cysts, four of which in the OFCD group, of which three children were moderately, one mildly and one severely affected; these cysts were included in the axial length for volumetric reasons.

**Table 5:** Growth or no growth of the affected eye.

Affected side	Follow‐up (years)	Growth unaffected/largest side	Growth affected/smallest side	Growth/no growth
PFV				
OS	5.2	2.30	0.20*	No
OS	3.5	4.80	5.00	Yes
OS	3.6	2.20	0.20	No
OD	1.8	2.70	0.80	Minimal**
OD	2.2	1.70	2.50	Yes
OD	4.0	3.10	0.90	Minimal
OS	2.6	2.40	−2.80	No
OS	1.3	3.50	3.10	Yes
OS	5.1	2.10	1.10	Minimal
OD	1.4	2.40	1.60	Minimal
COMB				
ODS	1.3	4.30	3.80	Yes/ yes
OS	2.8	0.40	0.90	Yes
OD	3.3	1.50	−8.20	No
OS	1.3	2.20	−4.10	No
OS	3.1	5.40	−2.70	No
OS	1.0	0.90	0.00	No
OD	2.8	0.90	−0.10	No
OS	2.2	1.90	1.90	Yes
ODS	3.0	3.50	2.80	Yes/ yes
SEC				
OS	3.8	0.90	−1.60	No
ASD				
ODS	5.0	3.30	6.20	Yes/yes
ODS	0.4	0.70	0.00	Yes/no
OD	1.4	1.10	0.80	Yes
OS	2.5	1.00	−0.80	No
OS	1.5	0.70	0.60	Yes
ODS	3.0	5.20	2.90	Yes/yes
OFCD				
ODS	0.8	1.60	2.30	Yes/yes
OD	3.5	2.30	4.50	Yes
OD	1.7	2.80	0.00	No
OD	2.0	4.00	−0.90	No
OS	1.6	1.40	0.40	Minimal
OS	5.1	1.30	1.10	Yes
OS	3.4	2.10	0.50	Minimal
OS	4.1	1.50	1.10	Yes
OD	2.2	1.20	1.90	Yes
OS	0.5	3.20	0.70	Minimal
REMNANT				
ODS	2.5	1.50	−2.00	Yes/no
OD	2.1	3.10	−2.20	No
OD	5.7	2.20	2.00	Yes
OD	3.8	0.60	3.80	Yes
OS	3.3	6.60	4.40	Yes

* Only changes of more than 0.2 mm were noted as possible growth.

** If growth was minimal compared to the growth of the healthy side, this was marked as ‘minimal’ growth.

In all cases with axial lengths below 45%, no apparent eye structure was seen during first examinations and ultrasonographic imaging and MRI showed either no structure at all, or only an ocular remnant. For all cases in the severe and moderate group, conformer treatment was started for expansive reasons because of disturbing asymmetry. In the mild group, conformer treatment was usually not deemed necessary for expansive reasons, but could be started to reconstruct facial appearance.

## Discussion

At present, no clear‐cut measurements are defined to indicate severity, and thus, treatment indication of MICA disorders and aetiological classifications does not guide us herein. The strength of our study is that we combine the clinical presentation of the MICA population with biometric data, both relevant for the assessment of the severity of MICA regarding treatment options. The most relevant biometric measurement to determine the severity of MICA is the rAL. Axial length (AL) measurements are easily performed using ultrasound by either a skilled ophthalmologist or radiologist in an outpatient setting. We found that rAL of the affected eye versus the contralateral unaffected eye indicated severe (0–45%), moderate (45–75%) and mild disease (75–100%), and this strongly correlated with rHPF and therefore subjected asymmetry. In our population, we aimed to start conformer treatment for the severe cases (rAL 0–45%) as soon as possible. In our experience, for moderate disease (rAL 45–75%) treatment is frequently indicated for asymmetry; for milder disease (rAL > 75%), this can be postponed until an older age.

In the severe group, imaging showed either an ocular remnant or no detectable ocular structure. As pure anophthalmia and cases with only an ocular remnant have a common feature of a cone‐shaped socket with the cone pointing to the orbital apex, a very small eyelid aperture, and no fornix formation, we collectively grouped these cases under ‘ocular remnant/anophthalmia’ (ORA). Most of these cases also showed some asymmetry of the brow shortly after birth, indicating a visible primary underdevelopment of the orbit. In our experience, these cases should be considered severe. In the moderately affected group, a normal socket anatomy existed, but with a microphthalmic eye with disturbing asymmetry for which treatment should be initiated within months of development. In the mild group, a slightly smaller AL or less obvious eyelid asymmetry was seen, for which reconstructive correction is possible, but expansive conformer treatment is unnecessary.

The severity classification in this paper is based on experiences with unilateral cases. This is done since comparison can be made to the healthy side, indicating the severity of the deformation. For asymmetric bilateral microphthalmic cases, the reference size should be the larger eye. The smaller eye could be treated when it is ruled out that it contributes to visual acuity. Bilateral ‘severe’ cases with no detectable eyes are a distinctive group since there is symmetry, however with no visual potential, severely underdeveloped sockets and bony hypoplasia. Because of the facial deformation, we advise to start treatment in an early stage (within a few weeks after birth), the same as for unilateral severe cases.

Cases within our clinical subgroups were randomly dispersed throughout the scatterplot (Fig. 1), suggesting that rAL measurements may be a better indicator for treatment necessity than aetiology classifications. However, clinical characteristics need to be taken into account.

It is important to distinguish between maldeveloped eyes with and without visual potential. The term nanophthalmos is sometimes used interchangeably with microphthalmos; for example, Warburg described this disorder as ‘simple microphthalmos’ (Warburg 1993). From a developmental point of view, there might be overlap between the two disorders (O'Grady 1971; Cross & Yoder 1976; Carifi et al. 2013; Steijns et al. 2013), although an important difference is that nanophthalmic eyes have a shortened, but furthermore intact anatomy of the eye. We regard this group as a different entity as it shows unaffected anatomy and visual potential. No cases of nanophthalmos were included in this study.

For clinical classifications, we used the most recent classification proposed by Skalicky (2013) and extended their non‐OFCD category as we experienced that phenotypes of PFV and ASD are critically different. Only a few of our PFV cases and more in the other subgroups showed reasonable growth of the affected eye. As a consequence, affected eyes may appear to be a mild MICA phenotype short after birth, but due to the lack of eye growth which may cause increasing asymmetry, they require treatment at a later stage. To the contrary, anterior segment disorders may present with normal or even enlarged axial lengths (Williamson & FitzPatrick 2014). In our population, ASD was frequently bilaterally affected (43%) with clinical abnormalities, although the fellow eye may have a normal axial length. Two of these bilateral cases presented with a mainly nontransparent cornea in the best eye, which spontaneously cleared for a minor part. As a result, these children showed visual acuity and interaction (1 year old) and easy navigation (3 years old). These examples denote a potential shortcoming of a cross‐sectional analysis for this group, as visual acuity is hard to determine at a young age and can change over time: some cases develop functional vision, while this is initially not determined as such. These cases must be handled with caution, and a (nontransparent) shell prosthesis or surgical intervention should not be initiated on the side with visual potential.

In any of the microphthalmic subgroups, some visual function of the affected eye may be present, even though this cannot always be explained. One case with an ocular remnant of 3 mm on one side and a minor ocular structure of 6.8 mm on the other side, without cornea, lens, and anterior chamber was treated with a transparent conformer to open the eyelids but showed signs of visual interaction with the environment being able to wave in response to a waiving person. We therefore emphasize the importance of careful follow‐up and guidance via low vision rehabilitation centres in these cases.

The published prevalence of extraocular abnormalities has a fairly large range of 33–95% (Kallen et al. 1996; Forrester & Merz 2006). We found serious abnormalities in 48% (28/58) of our cases. It is therefore of utmost importance that the child undergoes full physical examination. We found congenital cardiac disease to occur significantly more often in the OFCD group (4/12, Fisher's exact 0.014). One case was diagnosed with CHD7 mutation conforming CHARGE syndrome (Coloboma, Heart defects, choanal Atresia, Retardation (of growth and/or development), Genitourinary malformation and Ear abnormalities) (Hsu et al. 2014). Heart defects and MICA have been noted in several studies, but we do not see a clear relation with OFCD and urogenital abnormalities as described by Pasutto et al. (2007), Ragge et al. (2007), Skalicky et al. (2013), and Roos et al. (2016). Additionally, anatomical and functional central nervous system abnormalities (8/16, p = 0.001) and motor development delay (7/16, p = 0.006) occurred more often in ORA cases. One unilateral ORA case with an OTX2 mutation had an ectopic pituitary gland, an associated abnormality (Tajima et al. 2009; Dateki et al. 2010). In addition, OTX2 mutations have been found in anophthalmic cases with the absence of the optic nerve and chiasm. Although these structures are undeniably related, aetiological and clinical implications (such as follow‐up) for this finding are not clear at present.

Within our population, 47 cases were genetically tested of which 12/47 (25%) cases yielded a relevant mutation. All four of the bilateral ORA cases had a mutation in SOX2, identified as the most common cause for MICA up to now with varying percentages in the literature of 4.6–17.6% (Fantes et al. 2003; Ragge et al. 2005; Gerth‐Kahlert et al. 2013; Chassaing et al. 2014; Mauri et al. 2015). Clinical features are usually bilateral and may present with severe ‘anophthalmia’ with consistent extraocular symptoms such as brain malformations, motor abnormalities, axial hypotony, facial dysmorphism and dental anomalies, hypogenitalism in males, pubertal delay in females, postnatal growth failure and mental disability (Chacon‐Camacho et al. 2015). All our SOX2 cases presented with at least a few of these features. Furthermore, a relation between bilateral ocular anomalies and structural central nervous system anomalies have been indicated (Aktekin et al. 2005; Verma & Fitzpatrick 2007; Galindo‐Ferreiro et al. 2018); we however found these more often in unilateral cases (five) than in bilateral cases (two). Genetic mutations in PAX6 have not been identified within our population, which may be explained by the rarity of the mutation as observed in a study by Chassaing et al. (2014) who found the mutation only in 1 out of 150 patients. A recently published study with the largest cohort described in literature found consanguinity of the parents to be common (46.7% for 365 patients), indicating a potentially large role for genetics (Galindo‐Ferreiro et al. 2018).

In conclusion, our description of the clinical spectrum and biometric data extends the current classification systems. The use of severe, moderate and mild severity based on relative axial length measurements may aid in the decision to start orbital expansive treatment and may be used to compare different treatment strategies in the MICA spectrum.

## Supporting information


**Table S1.** Extraocular symptoms of different subgroups
