# Supplementary material for: Classification for treatment urgency for the microphthalmia/anophthalmia spectrum using clinical and biometrical characteristics
Source: Acta Ophthalmol. 2020 Feb 25;98(5):514–20. doi: 10.1111/aos.14364 (PMC7497250; doi:10.1111/aos.14364)
Supplement: Supplementary file 1 — Table S1. Extraocular symptoms of different subgroups [file AOS-98-514-s001.docx]

| **eTable 1. Extraocular symptoms of different subgroups** | | |
| --- | --- | --- |
| **Subgroup** | **Case no** | **Extraocular symptoms** |
| **Clinical anophthalmia** | 1 | motor development delay, growth deprivation |
|  | 2 | hypoplasia multiple cranial nerves (trigeminal, vestibulocochlear) leading to deafness, speech development delay |
|  | 21 | growth deprivation, ectopic pituitary gland without dysfunction |
|  | 22 | motor development delay, umbilical hernia, omphalocele, dysfunctioning ectopic pituitary, microcephaly |
|  | 26 | hypoplasia olfactorial nerve, speech delay with weak tongue and high-arched palate, skintag nose |
|  | 30 | motor development delay, hydrocephalus, encephalocele, epilepsy, cheilognathopalatoschisis |
|  | 35 | motor development delay, hypotony, extra teeth, no bladder control |
|  | 36 | hemilisencephaly, epilepsy, umbilical hernia |
|  | 47 | pectus excavatum, low IQ, hyperextension PIP joints |
|  | 57 | delayed teeth development |
|  | 61 | motor development delay, aphasia, hypotony, easily luxated patella's |
| **Optic fissure closure defects** | 18 | double outlet right ventricle and ventricle septum defect (Taussig-Bing syndrome) |
|  | 19 | motor development delay, persistent foramen ovale, weak pharynx, low weight and length |
|  | 23 | syndactyly dig 2-3 |
|  | 34 | axial hypotony, development delay |
|  | 37 | Wolf Hirschorn: coarctatio aortae, microcephaly, broad nose, thin lips, epicanthus, abnormal thumb implant left and rotation of end phalanx, short thumb right, long fingers, sandal gap, syndactyly dig 2-3 |
|  | 42 | esophageal atresia with tracheo-esophageal fistula, schizis palatum molle, apertura piriformis stenosis / choane atresia, atrial ectopic tachycardia, thoracic butterfly vertebra |
|  | 46 | kidney dysplasia, vesico-urethral reflux, hypertension, obesitas |
| **Combination** | 8 | naevus of OTA, isolated high FSH |
|  | 17 | panhypopituitarism, polydactyly, frontal bossing and multiple cerebral developmental defects with hypoplasia of the brain stem, tectum and corpus callosum |
|  | 40 | misshaped right ear with preauricular tag |
| **Persistent fetal vasculature** | 51 | motor development delay, patent ductus arteriosus, small length, bilateral middle ear atresia (no auditory canal and eardrums) therefore deafness |
|  | 55 | CLOVES syndrome: hypertrophic arm and feet, lymphangioma thorax, thoracal and lumbal scoliosis, interruption vena cava inferior, axial hypotony with peripheral hypertony |
|  | 58 | bifid uvula |
| **Anterior segment disorder** | 60 | cerebral disorders (partial agenesis corpus callosum, schizencephalia, polymicrogyria) |
|  | 29 | Gorlin syndrome: pulmonary stenosis, large skull diameter, hypertelorism, small nose, high forehead |
|  | 48 | motor development delay, double system left kidney |
| **Secondary** | 16 | coarctatio aortae, congenital diaphragmatic hernia |
